# Theory of mind, metacognition, and executive functions in adolescents with social anxiety disorder: a comparative study

**DOI:** 10.1186/s13034-025-00968-4

**Published:** 2025-10-08

**Authors:** Sefika Nurhuda Karaca Cengiz, Esra Guney, Ahmet Ozaslan, Esin Gokce Saripinar

**Affiliations:** 1 Department of Child and Adolescent Psychiatry, Eskişehir City Hospital, Eskişehir, Türkiye; 2 Department of Child and Adolescent Psychiatry, Gazi University Medical Faculty, Ankara, Türkiye

**Keywords:** Social anxiety disorder, Theory of mind, Executive function, Metacognition

## Abstract

**Background:**

Social anxiety disorder (SAD) in adolescence is associated with significant functional impairment and increased risk of chronic mental health difficulties. Recent research highlights the potential roles of theory of mind, executive functions, and metacognitive beliefs in the onset and maintenance of SAD; however, no study has yet evaluated these three cognitive domains simultaneously in a clinical adolescent sample.

**Methods:**

This was a cross-sectional descriptive study including 40 adolescents aged 12–16 years (SAD group: *M* = 14.45, *SD* = 1.48; control group: *M* = 13.89, *SD* = 1.32) who were diagnosed with SAD and 40 typically developing controls matched for age and sex. The participants completed the WISC-IV, the Reading the Mind in the Eyes Test (Eyes Test), the Faux Pas Recognition Test (FPRT), and the CNSVS subtests assessing Stroop Test, attention shifting, and continuous performance. The Metacognitions Questionnaire for Children and Adolescents (MCQ-C) was also administered. Parental reports provided sociodemographic data.

**Results:**

Compared to the control group, adolescents with SAD demonstrated lower performance in FPRT total scores, cognitive flexibility, and working memory, and reported higher MCQ-C total scores. Binary logistic regression analysis revealed that both working memory (β = −0.10, *p* < .05) and MCQ-C scores (β = 0.17, *p* < .01) were significant predictors of SAD diagnosis.

**Conclusions:**

These findings suggest that impairments in metacognitive beliefs and working memory may help distinguish adolescents with SAD from their typically developing peers. Incorporating these domains into clinical assessment and intervention strategies could enhance early detection and treatment outcomes.

## Background

Social anxiety disorder (SAD) is characterized by worrying about being judged or humiliated by others and marked by disproportionate fear and avoidance of social environments [1]. The symptoms of SAD often begin in childhood. In an epidemiological study conducted in Turkey, the prevalence of SAD among children was reported to range between 3% (without clinically significant functional impairment) and 1.55% (with impairment) [2]. Notably the diagnosis rate is higher at the end of adolescence, with a prevalence of approximately 10% [3, 4]. SAD, particularly during adolescence, can cause difficulties in peer interactions, contribute to academic problems, and lead to increased school absenteeism [5, 6]. SAD that starts early or is untreated in childhood and adolescence poses a risk of continuing into adulthood [7]. It has been reported that the functionality of adults who do not receive treatment is more impaired due to comorbidities, and they are less inclined to engage in life events such as establishing close relationships, marrying, and having children [8, 9]. These findings underscore the importance of early identification and targeted interventions for SAD, given its potential to disrupt developmental trajectories and impair long-term functioning. In recent years, theory of mind (ToM), metacognition, and executive functions have been recognized as notable cognitive processes associated with SAD. Targeting these higher-level cognitive processes may support more effective and sustainable treatment outcomes [10–13].

### Theory of mind and social anxiety disorder

ToM -the ability to reason accurately about the beliefs, intentions, desires, and emotions of others- is necessary for a successful social life and interpersonal relationships [14, 15]. Although various classifications for ToM exist, the most common are social-cognitive and social-emotional [16]. Evaluating the mental state of others on the basis of observable information, such as eyes or facial expressions, is defined as the social-emotional component. The ability to interpret the actions of others or reason about these actions is defined as the social-cognitive component. ToM has been studied in various psychiatric conditions such as autism spectrum disorder, obsessive-compulsive disorder, attention-deficit/hyperactivity disorder, depression, and SAD [15, 17–22].

Clark and Wells posited that impairments in *mentalization*, understanding and reflecting on one’s own and others’ mental states, may contribute to the development and maintenance of SAD [6, 23]. When individuals struggle to identify and interpret others’ emotions and intentions accurately, they may experience increased anxiety in social contexts. Individuals with SAD are not considered entirely deficient in ToM; rather, they tend to attribute exaggerated and context-inappropriate meanings to others’ emotions in social situations. Therefore, assessing ToM abilities may hold clinical significance for individuals with SAD [11]. Alvi et al. found that social anxiety was negatively associated with higher-level social cognitive abilities, including ToM and empathic accuracy, especially in response to positive stimuli [24]. In a longitudinal study conducted in the United States with a community sample of 152 children aged 3 to 6 years, stronger ToM abilities were found to be associated with a decreased risk of developing social anxiety later in childhood [25]. Similarly, another study reported that better ToM performance predicted lower social anxiety levels and fewer instances of peer rejection after one year [26]. In line with these findings, a community-based study identified a moderate link between social cognitive task performance and social anxiety, emphasizing the need for interventions targeting this domain [27]. A recently published meta-analysis involving a large sample of children and adolescents aged 4 to 19 revealed a small but significant negative association between anxiety and ToM performance. This association appeared stronger for affective ToM domains than for cognitive ones [28]. Taken together, exploring ToM in adolescents with SAD may inform tailored treatment approaches and contribute to a deeper understanding of its cognitive foundations.

### Metacognition and social anxiety disorder

Metacognition is a system that enables individuals to be aware of the events and processes in their minds, and to manage them appropriately [29]. The metacognitive model argues that mental disorders arise from dysfunctional metacognition rather than negative thoughts [30]. According to the metacognitive model, psychiatric disorders are associated with a particular pattern of thinking called the cognitive attention syndrome (CAS) [31]. This syndrome includes worry, rumination, a heightened focus on potential threats, and ineffective coping mechanisms, which are thought to be central to the development and persistence of these disorders. Wells suggested that metacognitive beliefs play a critical role in the onset and maintenance of various psychological disorders, including social anxiety [30, 31]. Metacognitive beliefs are prevalent in the persistence of social anxiety, heightening individuals’ threat perceptions and triggering self-focused attention and ruminative thinking. Metacognition has a significant effect on social anxiety and may be independent of cognition [32]. This assumption is supported by studies reporting the positive effects of metacognitive interventions, rather than interventions targeting social phobic beliefs and schemas, in the treatment of SAD [33–35]. In a recent study evaluating the interrelationship between metacognition, cognition, and social anxiety, metacognitive beliefs were able to predict both social interaction anxiety and social self-beliefs. Negative social self-beliefs may be a product of metacognition, and therefore, metacognitive beliefs should be a central focus in treating SAD [10]. Notably, a study of children diagnosed with anxiety disorders showed that the presence of negative metacognition and multiple diagnoses predicted the diagnosis of SAD [36]. Similarly, a study conducted with children aged 7–13 years who were diagnosed with SAD and generalized anxiety disorder, found a significant difference in metacognitive processes [37]. A review of the literature indicates that, although a limited number of studies have focused on metacognitive processes in adults, research specifically addressing adolescents remains notably scarce. Considering that the onset of SAD frequently coincides with adolescence due to its developmental characteristics, exploring metacognition within this population could offer valuable insights and enhance the effectiveness of interventions.

### Executive function and social anxiety disorder

In anxiety disorders, the balance between top-down attention control and bottom-up stimulus-driven attention is disrupted, increasing sensitivity to negative environmental cues [38]. Those affected by SAD may have difficulty regulating their attention effectively during social interactions due to intense anxiety. As a result, they tend to focus more on internal cues (e.g., heart palpitations, sweating) rather than external social signals. They may also engage in maladaptive mental imagery and hypothetical scenarios, which can amplify the perceived threat in social contexts. These self-focused and distorted cognitive processes are considered key factors in the maintenance of social anxiety [39–41]. Fujii et al. reported that SAD may be accompanied by executive dysfunction [42], although it is not considered an etiological factor for the disorder. In a transdiagnostic study comparing autism, early psychosis, and social anxiety disorder, individuals with social anxiety reported impairments in executive functions on self-report measures, while their performance on objective cognitive tests remained within the normal range [43]. A longitudinal study by Morea and Calvete (2021) found a reciprocal relationship between cognitive flexibility and social anxiety, suggesting that difficulties in one may predict worsening in the other over time [44]. Moreover, working memory-based interventions have been found to be effective in reducing SAD symptoms [13]. In light of studies evaluating executive functions—particularly working memory and cognitive flexibility—in children and adolescents with SAD, addressing difficulties in these domains may enhance clinical outcomes for those who exhibit such challenges.

### Neurodevelopmental context

Adolescence is a critical developmental window marked by substantial neurobiological changes in socio-affective and motivational brain systems, making it a high-risk period for SAD onset. This vulnerability is influenced by both early-life temperamental traits, such as behavioral inhibition, and neurodevelopmental responses to social evaluation and reward processing during adolescence [45]. Neuroimaging studies have consistently implicated a network of brain regions, including the prefrontal cortex, amygdala, and insula, in the pathophysiology of SAD, all of which play essential roles in threat detection, interoceptive awareness, emotional reactivity, and the evaluation of social stimuli [46–48].

Within this context, deficits in higher-order cognitive domains such as ToM, metacognition, and executive functioning are hypothesized to share overlapping neurobiological substrates with SAD. Although ToM involves distributed neural systems, mental state attribution to oneself and others has been particularly linked to limbic and paralimbic regions such as the amygdala, orbitofrontal cortex, ventromedial prefrontal cortex, and anterior cingulate cortex as well as prefrontal subregions including the dorsomedial and inferolateral cortices [49].

Moreover, studies have shown that executive function deficits observed in individuals with SAD involve dysfunctions in brain regions such as the prefrontal cortex, insula, amygdala, and precuneus [50] Similarly, metacognitive impairments frequently reported in SAD patients have been linked to altered functioning in regions involved in self-awareness and internal experience. These include the anterior insula and dorsal anterior cingulate cortex [51, 52], as well as the medial prefrontal cortex and precuneus [53].

What ToM and metacognition have in common is the recognition of one’s own and others’ emotions and mental states. The cognition model explained in ToM is considered “metacognitive knowledge” [54], and ToM and executive function development are thought to be linked [55]. This is supported by evidence that the frontal lobe, alongside other relevant regions, is actively involved in both ToM and executive functioning, with this relationship observed across clinical populations [56]. Executive functions and metacognition may play an essential role in cognitive development and follow similar developmental timelines. The interaction between these constructs is considered dynamic. Both executive functions and metacognitive abilities may contribute integratively to an individual’s capacity to form and apply meta-representations related to thinking and learning processes. Although this was not the central emphasis of the present study, some scholars have proposed that ToM may be involved in this integrative framework as it pertains to understanding one’s own and others’ mental states, including intentions, beliefs, and cognitive strategies [57].

### Study rationale and aim

Theoretically, these three cognitive domains are thought to influence one another and collectively shape social functioning. ToM involves understanding others’ intentions; executive functions refer to processing this information and regulating behavior accordingly; and metacognition encompasses the awareness and evaluation of these processes. Recent studies in adolescents with SAD suggest associations between these domains; however, no study to date has examined all three within the same sample. To address this gap, the present study aims to simultaneously evaluate ToM, metacognitive abilities, and executive functioning in adolescents with SAD, and determine which of these domains may serve as cognitive predictors distinguishing them from their typically developing peers.

## Methods

### Participants

The participants in this study were selected from individuals who were admitted to the Department of Child and Adolescent Mental Health and Diseases at Gazi University Faculty of Medicine between October 2021 and October 2022. The clinical group consisted of adolescents aged 12 to 16 who were diagnosed with SAD on the basis of clinical interviews and the Schedule for Affective Disorders and Schizophrenia for School-Age Children – Present and Lifetime Version, DSM-5 November 2016 Turkish Adaptation (K-SADS-PL-DSM-5-T), which was administered by the researcher.

The inclusion criteria for the clinical group were: not having received any treatment for SAD or taking any psychiatric medication in the past month, obtaining a score of 80 or above on the WISC-IV intelligence test, and not meeting the criteria for any additional psychiatric diagnoses. The exclusion criteria encompassed individuals with co-occurring psychiatric disorders, use of psychoactive medications within the past month, presence of neurological or metabolic conditions, or a full-scale IQ score below 80 on the WISC-IV.

The control group comprised 40 children and adolescents who applied to the outpatient clinic for counseling services, who demonstrated good academic performance, who were not diagnosed with any psychiatric disorder through clinical interview and K-SADS-PL-DSM-5-T, and shared similar sociodemographic characteristics with the clinical group. The same inclusion and exclusion criteria applied to this group as well.

All procedures were conducted in accordance with the ethical standards of the institutional research committee and the Declaration of Helsinki and its subsequent amendments. Informed consent was obtained from all participants and their parents prior to participation. During the course of the study, two adolescents from the SAD group who had initially met inclusion criteria and provided verbal and written informed consent, along with five participants from the control group, were excluded upon their request to withdraw from the study.

### Data collection tools and procedure

#### The schedule for affective disorders and schizophrenia for school-age children present and lifetime version, Dsm-5 November 2016 -Turkish adaptation (K-SADS-PL-DSM-5-T)

The Turkish adaptation of this semi-structured interview schedule, originally developed by Kaufman et al. [58] and updated in line with DSM-5 diagnostic criteria, was finalized as the K-SADS-PL-DSM-5-T. The Turkish validity and reliability study was conducted by Ünal et al., who reported satisfactory who reported that the instrument demonstrates high inter-rater agreement, test–retest reliability, and internal consistency across diagnostic modules [59].

### Wechsler ıntelligence scale for children-fourth edition (WISC-IV)

The Wechsler Intelligence Scale for Children, Fourth Edition (WISC-IV), developed by Wechsler, is designed to assess the cognitive abilities of children aged 6 to 16 years and 11 months [60]. The scale comprises ten core and five supplementary subtests, yielding four index scores—Verbal Comprehension, Perceptual Reasoning, Working Memory, and Processing Speed—as well as a Full Scale Intelligence Quotient (FSIQ). The FSIQ ranges from 40 to 160, with a mean of 100 and a standard deviation of 15; higher scores reflect stronger intellectual functioning. The Turkish adaptation and standardization of the WISC-IV was conducted by Karakaş et al. (2011), and the scale has demonstrated satisfactory psychometric properties in Turkish samples [61].

### The “reading the mind in the eyes test” (Eyes Test)

Baron-Cohen et al. (1997) developed the Eyes Test to measure social-emotional skills in children with Asperger syndrome and autism [62]. In the Eyes Test, the participant is asked to look at each pair of eyes and check the option that best describes what the people in the photograph are thinking or feeling. Each question has only one correct answer, and the number of questions answered correctly is calculated in the evaluation. The maximum score that can be obtained from the test is 28. A high score indicates good social cognition and ToM skills. This study used the Turkish child version of the Eyes Test developed by Girli (2014), which is intended for children aged 6 to 16. The Turkish version demonstrated acceptable internal consistency (Cronbach’s α = 0.72) and construct validity across age, gender, and diagnostic groups [63].

### Faux pas recognition test-child version (FPRT)

FPRT was developed by Baron Cohen and colleagues (1999) to assess higher ToM skills [64]. In the original study of the FPRT, ten faux pas stories, and ten control stories were randomly placed. The practitioner reads the stories in order, and the participants’ responses are recorded and scored. A minimum score of zero and a maximum score of twenty is obtained from the whole test, and an increase in score indicates an increase in ToM skills. The validity and reliability study of the Turkish version for children aged 9 to 17 was conducted by Şahin et al. (2020). The study included a sample of 224 children, consisting of 127 diagnosed with ADHD and 97 typically developing peers. The test demonstrated adequate test–retest reliability over a 4-week interval and was found to significantly differentiate between clinical and control groups [65].

### Cns vital signs (CNSVS)

CNSVS is a computer-assisted neuropsychological test battery that can be applied to individuals between the ages of 7 and 89 and whose validity and reliability were established by Gualtieri and Johnson [66]. It initially consisted of seven tests: verbal and visual memory, finger tapping, symbol number coding, Stroop Test, attention shift test, and a continuous performance test. Later, three additional tests were added: the perception of emotions test, the non-verbal reasoning test, and the four-part continuous performance test. Each test has its own sub-scores, and main section scores are calculated using the sub-scores [67]. The test offers the opportunity to examine various main sections separately by creating different combinations. The Stroop Test, the Attention Shifting Task, and the Continuous Performance Test were applied since our study aimed to assess working memory and cognitive flexibility.

### The metacognition questionnaire for children and adolescents (MCQ-C)

The Metacognitions Questionnaire for Children (MCQ-C) was developed by Bacow et al. (2009) based on the theoretical framework of Cartwright-Hatton and Wells’ metacognitive model. This version was specifically adapted for children by simplifying item content and removing developmentally inappropriate subscales. MCQ-C scale consists of 24 items and four subtests: positive meta-worry, negative meta-worry, cognitive monitoring, and a fourth related to superstition, punishment, and responsibility. Scores are calculated for each subscale as well as a total scale score. Each item in the scale is answered on a four-point Likert-type rating scale starting with “ [1] strongly disagree” and ending with “ [4] strongly agree.” Potential scores range from 24 to 96, and higher scores indicating more dysfunctional or maladaptive metacognitive beliefs [68]. The Turkish validity and reliability study was conducted by Irak (2012) who reported acceptable internal consistency (Cronbach’s α = 0.81) and satisfactory construct validity [69].

### Procedure

After providing a verbal explanation of the study’s objectives and methodology, the researcher administered the sociodemographic data form to the parents of both the clinical and control group participants who agreed to participate. In the clinical group, adolescents aged 12 to 16 years were first assessed through a semistructured clinical interview based on the K-SADS-PL-DSM-5-T, conducted by a child and adolescent psychiatrist. The WISC-IV intelligence test was administered by a licensed clinical psychologist. Only those with no additional psychiatric diagnosis and with a full-scale IQ score of 80 or above were included in the study. In the control group, the same diagnostic interview and WISC-IV assessment were applied under the same conditions.

Approximately 20 min after the completion of the WISC-IV, the remaining assessments were conducted by the researcher in a single session lasting approximately 75–90 min, depending on the participant’s pace. During this session, the CNSVS computerized test battery was administered, followed by the FPRT and the Eyes Test, with 10-minute breaks in between. Finally, participants were asked to complete the MCQ-C as a self-report measure.

### Statistical analysis

The data obtained in this study were analyzed with the SPSS 22.0 program. The study’s data were initially analyzed descriptively, employing frequency and percentage distributions. In addition, the similarity of the distributions of adolescents diagnosed with SAD and adolescents in the control group was checked by Pearson’s chi-square and independent groups t-test. Pearson’s correlation analysis was used to examine the association between dependent variables. A binary logistic regression was conducted to assess the impact of demographic and cognitive factors on the likelihood of participants receiving a diagnosis of SAD. The significance level was set as *p* < .05 when findings were interpreted.

### Ethics committee approval

The Ethics Commission of Gazi University approved this research at its meeting on 03.08.2021 and was numbered 12.

## Results

In the study, 30 girls and 10 boys participated in each of the SAD and control groups. Sociodemographics are provided in Table 1*and* Table 2. There were no significant differences between the SAD and control groups in terms of gender, parental education, or employment status. Full-scale IQ scores based on WISC-IV were also compared between groups, and no significant difference was observed (Table 3).

**Table 1:** Age distribution of participants

	Mean (SD)	Range	t	sd	*p*
SAD	**14.45 (1.48)**	**12–16**	1.79	78	0.08
Control	**13.89 (1.32)**	**12–16**			

**Table 2:** Comparison of sociodemographic characteristics of SAD and control groups

Variable	Category		SAD *n* (%)	Control *n* (%)	$\:{\varvec{\chi\:}}^{2}$	*p*
Child’s Sex	Girl		30 (75)	30	0	1
	Boy		10 /25)	10		
Education Level of Mother	Primary School		5 (12,5)	2 (5,1)		
	Secondary School		3 (7,5)	0 (0)		
	High School		13 (32,5)	14 (35,9)	4.69	0.19^1^
	University		19 (47,5)	23 (59)		
Education Level of Father	Primary School		6 (15)	1 (2,6)		
	Secondary School		1 (2,5)	1 (2,6)		
	High School		18 (45)	11 (28,9)	9.31	0.062^1^
	University		15 (37,5)	25 (65,8)		
Employment Status of Mother	Employed		26 (65)	21 (53,8)		
	Unemployed		13 (32,5)	18 (46,2)	2.33	0.31^2^
	Retired		1 (2,5)	0 (0)		
Employment Status of Father	Employed		37 (92,5)	36 (94,7)		
	Unemployed		0 (0)	1 (2,6)	1.36	0.37^2^
	Retired		3 (7,5)	1 (2,6)		

^1^Independent t-test

^2^Chi-squared test

**Table 3:** Comparison of Eyes Test, FPRT, MCQ-C scores, and terms of CNSVS domain scores of participants in the SAD and control groups

	Group	Mean (S.d.)	t	df	*p*
Total Eyes	SAD Control	20.75(2.13) 21.58(1.99)	-1.79	78	0.077
FPRT Total Scores	SAD Control	16.05(2.14) 17.45(1.38)	-3.49	78	0.001**
MCQ-C Total Score	SAD Control	67.80(8.57) 52.93(11.28)	6.64	78	0.001**
Cognitive Flexibility	SAD Control	95.40(2.72) 103.95(1.42)	-2.78	78	0.007**
Working Memory	SAD Control	96.70(1.92) 106.98(1.69)	-4.03	78	0.001**
WISC- IV (FSIQ)	SAD Control	100.70 (10.80) 105.23(11.86)	-1.78	78	0.08

* <0.05; ** <0.01

Table 3 presents the comparison of Eyes Test total scores, FPRT total scores, MCQ-C total scores, and CNSVS cognitive flexibility, and the working memory between participants in the SAD and control groups. No differences were detected between the groups in Eyes Test scores. A statistically significant difference was observed in FPRT total scores (t=-3.49, *p* < .01). The SAD group’s MCQ-C total scores were found to be higher than those of the control group.

Table 4 includes the results of binary logistic regression analysis. Variables showing significant differences between the two groups in the t-test were further evaluated using logistic regression analysis. Univariate predictors, adjusted for age and sex, were analyzed using binary logistic regression. The results showed that metacognition scores (β = 0.17, *p* < .01) and working memory (β = − 0.10, *p* < .05) were found to predict the SAD significantly.

**Table 4:** Binary logistic regression analysis on social anxiety disorder

Variable	β	Sig.	Exp(B)	95% CI for Exp(B)	95% CI for Exp(B)
				Lower	Upper
Age	0.399	0.151	1.491	0.864	2.571
Sex/Boys	0.76	0.401	2.138	0.363	12.613
FPRT Total Score	-0.418	0.084	0.659	0.41	1.057
Eyes Total Score	-0.329	0.105	0.72	0.483	1.072
MCQ-C Total Score	0.17	0.001**	1.186	1.089	1.291
Cognitive Flexibility	0.028	0.464	1.028	0.955	1.107
Working Memory	-0.108	0.01*	0.898	0.827	0.974

* <0.05; ** <0.01, CI: confidence interval B: non-standardized coefficients; β: Standard coefficients;

Pearson correlation analyses were conducted separately for the SAD and control groups to examine associations among the key study variables. The results are presented in Tables 5 and 6. In the SAD group, executive function measures were significantly correlated with WISC-IV scores. However, no meaningful associations were found between MCQ-C Total Score, FPR Total Scores, and the other core variables. A similar pattern was observed in the control group, where executive functions were associated with WISC-IV, but no other strong correlations emerged.

**Table 5:** Correlations among key variables in the SAD group

		FPRT Total Scores	Cognitive Flexibility	Working Memory	MCQ-C Total Score	Total Eyes	WISC- IV (FSIQ)
FPRT Total Scores	r		0.253	0.215	-0.046	0.082	0.300
	df		38	38	38	38	38
	p		0.115	0.84	0.780	0.617	0.060
Cognitive Flexibility	r	0.253		0.507***	-0.159	0.311	0.498**
	df						
	p	0.115		< 0.001	0.328	0.051	0.001
Working Memory	r	0.215	0.507***		0.021	0.034	0.332*
	df						
	p	0.184	< 0.001		0.899	0.836	0.036
MCQ-C Total Score	r	0.-0.046	-0.159	0.021		0.086	0.004
	df						
	p	0.780	0.328	0.899		0.600	0.982
Total Eyes	r	0.082	0.311	0.034	0.086		0.278
	df	38	38	38	38		38
	p	0.617	0.051	0.836	0.600		0.082
WISC- IV (FSIQ)	r	0.300	0.498	0.332*	0.004	0.278	
	df						
	p	0.060	0.001	0.036	0.982	0.082	

**Table 6:** Correlations among key variables in the control group

		FPRT Total Scores	Cognitive Flexibility	Working Memory	MCQ-C Total Score	Total Eyes	WISC- IV (FSIQ)
FPRT Total Scores	r		0.175	0.079	0.060	0.156	-0.042
	df		38	38	38	38	38
	p		0.280	0.626	0.713	0.336	0.795
Cognitive Flexibility	r	0.175		0.352*	0.189	0.128	0.394*
	df	38		38	38	38	38
	p	0.280		0.026	0.243	0.433	0.012
Working Memory	r	0.079	0.352*		-0.017	-0.036	0.569***
	df	38	38		38	38	38
	p	0.626	0.026		0.916	0.827	< 0.001
MCQ-C Total Score	r	0.060	0.189	-0.017		0.008	0.067
	df	38	38	38		38	38
	p	0.713	0.243	0.916		0.962	0.683
Total Eyes	r	0.156	0.128	-0.036	0.008		0.158
	df	38	38	38	38		38
	p	0.336	0.433	0.827	0.962		0.331
WISC- IV (FSIQ)	r	-0.042	0.394*	0.569***	0.067	0.158	
	df	38	38	38	38	38	
	p	0.795	0.012	< 0.001	0.683	0.331	

* *p* < 0.05, ** *p* <0.01, *** *p* <0.001

## Discussion

This study aimed to examine executive functions, ToM skills, and metacognitive functions, as well as the relationship among these variables in adolescents diagnosed with SAD. Participants with SAD demonstrated lower performance in faux pas recognition, cognitive flexibility, and working memory. They scored significantly higher on the metacognitive beliefs questionnaire, indicating more maladaptive metacognitive patterns compared to the control group. However, no significant group difference was found in Reading the Mind in the Eyes Test scores. After controlling for age and sex, only working memory and metacognitive beliefs emerged as significant predictors of SAD diagnosis. These findings underscore the potential relevance of higher-order cognitive functions in the developmental trajectory and functional impairment associated with SAD.

### Theory of mind and social anxiety disorder

ToM was assessed using the FPRT and the Eyes Test. While group differences were observed in FPRT scores, no significant differences emerged in Eyes Test scores. Furthermore, FPRT performance did not significantly predict SAD diagnosis after controlling for age and sex.

Previous studies have reported lower Eyes Test scores in adolescents and adults with SAD compared to controls [17–19]. For instance, in a study comparing adults with SAD, major depressive disorder, and healthy controls, individuals with SAD exhibited poorer Eyes Test performance [18]. In contrast to these findings, our study did not reveal any significant differences in Eyes Test scores between groups. A possible reason for this discrepancy is that, unlike prior studies, our sample included only adolescents whose cognitive abilities were objectively assessed and who had no comorbid diagnoses or prior treatment history. This methodological approach may have reduced variability in test performance and contributed to different outcomes.

In our study, although FPRT scores were lower in adolescents with SAD compared to the control group, they were found to be nonpredictive of SAD diagnosis after controlling for age and sex differences. Prior studies using the FPRT in individuals with SAD is limited. One study reported no significant difference between individuals with SAD and the control group in terms of faux pas recognition; however, the same study identified the omission of differences in medication use and treatment status between the groups as a limitation [18]. Our study minimized such confounding factors by including only untreated adolescents and screening for normal cognitive functioning. Furthermore, participants had adequate cognitive functioning, as verified by WISC-IV scores. Given that faux pas recognition engages cognitive functions like working memory and attention, the reduced performance observed in the SAD group may reflect subtle ToM impairments that influence social misinterpretations and increase perceived social threat.

While the Eyes Test assesses emotional ToM, the FPRT captures both cognitive and emotional aspects. Studies involving both measures have shown ToM impairments across various psychopathologies [70, 71] yet few have specifically examined both in adolescents with SAD. Our results suggest that the cognitive component of ToM may be more sensitive to SAD-related impairments during adolescence. This finding may reflect adolescents’ challenges in cognitively interpreting complex social situations. Failure to recognize social subtleties such as faux pas could reinforce distorted beliefs about being judged or misunderstood, thus perpetuating social anxiety.

Contrary to our initial hypothesis, ToM measures did not independently predict SAD diagnosis. This may indicate that ToM deficits do not directly underlie SAD or that their role is moderated by other cognitive or emotional processes. Methodological factors such as the sensitivity of the tools used, sample size, or developmental characteristics of participants may also account for this outcome.

These results point to the need for future research with larger and more diverse samples, using refined tools that separately assess both the cognitive and the affective components of ToM. Given the developmental transitions that occur during adolescence, longitudinal studies are also recommended to clarify how the relationship between ToM and SAD evolves over time. In addition, the use of more comprehensive ToM assessment batteries in future research may provide a more nuanced understanding of ToM impairments in adolescents with SAD

### Metacognition and social anxiety disorder

In our study, revealed that adolescents with SAD differed from their peers in metacognitive beliefs, which significantly predicted SAD diagnosis even after controlling for age and sex. Prior studies that comparing children with SAD to their peers with typical development reported differences in metacognitive beliefs, consistent with our findings [72, 73]. Simiarly, Esbjørn et al. (2021) also established the fact that children aged between seven to thirteen years and diagnosed with SAD expressed higher metacognitive scores than the participants of the control group, whereas processes of metacognition served as the predictors of SAD.

Our findings provide support for the Self-Regulatory Executive Function [74], which posits that negative beliefs about the uncontrollability and danger of one’s thoughts, along with the perceived consequences of failing to control these thoughts, are fundamental to the development and maintenance of clinical anxiety and depression. Positive metacognitive beliefs in the model activate the CAS, whereas negative beliefs enhance perceptions of threat, hinder adaptive strategies for managing these threats, and prolong CAS activity, thereby perpetuating emotional distress [75].

Taken together, our results highlight a specific and clinically meaningful role of metacognitive beliefs in SAD. Targeting metacognitive beliefs through interventions—such as metacognitive therapy—may enhance treatment outcomes in adolescents with SAD. This approach may be particularly relevant during adolescence, a developmental period marked by heightened self-focus and emerging metacognitive awareness. One recent study suggested that metacognitive beliefs predict both social interaction anxiety and social self-beliefs, and therefore should be the central treatment target in SAD [10]. Longitudinal studies are particularly needed to evaluate whether changes in metacognitive beliefs correspond with reductions in CAS activity and improvements in social anxiety symptoms over time

### Executive function and social anxiety disorder

In our study, individuals with SAD exhibited significantly lower performance in both cognitive flexibility and working memory compared to the control group. Additionally, working memory emerged as a significant predictor of SAD diagnosis, even when controlling for age and sex.

The literature on cognitive flexibility in SAD has yielded mixed findings. One study reported that individuals with SAD performed worse on attention-shifting and continuous performance tests [43]. Another study conducted with adults without comorbidities found that executive dysfunction was associated with greater social anxiety severity [42]. However, a review article assessing neuropsychological performance in SAD patients revealed that only one out of five studies using set-shifting tests reported significant impairments [76]. While some studies investigating the relationship between anxiety and executive function reported that social anxiety symptoms predict improvements in cognitive flexibility, others reported that anxiety impairs cognitive flexibility by negatively affecting the attention system [38, 44]. A longitudinal study involving 689 adolescents found that increased social anxiety predicted improvements in cognitive flexibility over a one-year period [44]. This contrasts with our findings, likely owing methodological differences: while that study involved a community sample without a formal SAD diagnosis, our study included clinically diagnosed adolescents, assessed through structured interviews.

These findings suggest that working memory is an effective executive function in adapting to new situations and maintaining tasks. Individuals with social anxiety symptoms may experience impairments in their executive functions because they are affected by the distracters [77]. Compared with that in the control group, impaired working memory was observed in adults with SAD, and this impairment was associated with the severity of social anxiety [78]. In our current study, working memory was found to show lower performance in individuals with SAD according to the literature. Working memory was found to predict the diagnosis of SAD when age and sex were equalized. According to our findings, when working memory performance is impaired, the diagnosis of SAD will persist even if cognitive flexibility is not affected. Considering the findings of our study, it is suggested that clinicians should recognize the importance of assessing working memory in adolescents as part of the diagnostic process for SAD. In addition to the findings of our study, although it has been reported that SAD treatment improves working memory [13], follow-up studies are essential to establish cause-effect relationships during developmental stages and evaluate treatment responses

Executive and metacognitive functions follow similar developmental trajectories, and both play crucial roles in enabling individuals to adapt to novel and challenging tasks [57, 79]. In our study, the parameters predicting the diagnosis of SAD were identified as metacognition and working memory. The literature includes studies indicating that working memory and metacognition jointly influence learning and problem-solving abilities [80, 81]. Our findings may suggest that working memory is more closely associated with metacognition than other executive functions. In light of our results, evaluating metacognitive functions and working memory may be considered particularly valuable for the diagnostic assessment of SAD in children and adolescents, as well as for developing treatment strategies and addressing treatment-resistant cases.

### Strengths and limitations

One notable strength of the present study is that, to our knowledge, it is the first, to our knowledge, to evaluate ToM skills, executive functions and metacognitive functions simultaneously in adolescents with a clinical diagnosis of SAD. This multidimensional assessment offers a more comprehensive understanding of higher-order cognitive processes associated with SAD during adolescence. Another methodological strength lies in the administration of the WISC-IV intelligence test across both the clinical and control groups to ensure that general cognitive functioning was within a comparable and normative range. The lack of significant differences in WISC-IV scores supports the validity of the group comparisons on ToM, executive function, and metacognition, as differences cannot be attributed to overall cognitive ability. The study further benefits from well-matched sociodemographic characteristics between groups, including age, sex, and educational background, which enhances internal validity. In addition, this is the first study to utilize the CNSVS -a computerized, standardized neuropsychological battery- to assess executive functions in adolescents with SAD. The objectivity and precision offered by computerized assessments reduce observer bias and improve measurement sensitivity. When evaluating ToM, both social-emotional (Eyes Test) and social-cognitive (Faux Pas Recognition Test) domains were measured, enabling a nuanced assessment of different components of social cognition. A further strength lies in the strict inclusion criteria: only adolescents with a confirmed diagnosis of SAD, no comorbid psychiatric disorders, and no prior treatment history were included. This approach enhances the specificity and clinical interpretability of the results.

Despite these strengths, the lack of significant associations between metacognition, executive functions, and ToM in our study may be attributable to several factors. First, the types of measurements used may have influenced the results. Executive functions were assessed through performance-based tasks, while metacognition was evaluated via self-report, and ToM was measured through structured tasks. These differing modalities may tap into distinct levels of cognitive awareness and metarepresentational capacity. Moreover, variations in response formats and cognitive demands across these assessments may have restricted the shared variance among constructs, potentially weakening the expected correlations. Second, the associations among these constructs may be stronger in clinical samples where impairments are more pronounced and interdependent. Although our study included adolescents diagnosed with SAD, the sample may have been heterogeneous in terms of symptom severity and cognitive profiles. This variability might have contributed to the weak or non-significant associations observed. Furthermore, the relatively small sample size in our study may have limited the statistical power to detect subtle associations between cognitive variables. Therefore, the absence of significant correlations should be interpreted with caution and not be generalized as evidence of no underlying relationships. While mediation or path analyses could theoretically explore the relationships between these constructs, the lack of significant bivariate correlations in the present dataset did not provide a sufficient foundation for robust modeling. Future studies incorporating more homogeneous clinical subgroups or longitudinal designs may better clarify the temporal and functional dynamics among these cognitive domains.

This study has several limitations. The sample size was relatively small, and the age of symptom onset was not assessed. Owing to the lack of retrospective evaluation, it remains unclear whether deficits in ToM, executive functions, and metacognitive abilities precede or follow the onset of the disorder. Furthermore, the majority of participants in the clinical group were female. While this gender imbalance might be viewed as a limitation, it actually aligns with broader epidemiological patterns. In SAD prevalence studies conducted in various community samples, it has been reported that the prevalence of SAB is higher in girls compared with boys [3, 82–84]. Though large-scale prevalence data from Turkey remain limited, clinical samples show similar trends. For instance, a two-stage urban-based study in Istanbul reported that female gender was significantly associated with social anxiety levels in clinical interviews [85] Thus, the gender distribution observed in our sample likely reflects well-established epidemiological patterns seen in clinical populations, rather than sampling bias. Nevertheless, future studies with more balanced gender representation would help clarify whether the cognitive correlates of SAD differ by sex. In addition, because the study employed a cross-sectional design, causal relationships could not be established, nor could the impact of these cognitive functions on treatment outcomes be evaluated in the absence of follow-up assessments. Another limitation relates to the selection of the control group. Although the participants did not meet diagnostic criteria for any psychiatric disorder, they were recruited from individuals who sought consultation at a clinical setting. Therefore, the possibility of subthreshold psychiatric symptoms cannot be ruled out. This may have influenced the findings and limits their generalizability to truly nonclinical populations.

Moreover, it should be acknowledged that uncontrolled contextual and individual factors such as social experiences, family interaction patterns, and developmental background may have influenced the observed associations. To better account for such variables, future research is encouraged to incorporate multi-informant data sources, including parent, teacher and clinician-based reports. Finally, the modest sample size limited the use of more advanced statistical methods. More sophisticated modeling approaches—such as mediation or path analysis—may yield deeper insight into the interplay of cognitive mechanisms if applied to larger samples. Future longitudinal studies should examine whether changes in metacognitive beliefs and executive functioning predict the onset, progression and treatment response of SAD. Additionally, experimental studies specifically targeting executive functions or metacognition may further clarify the causal roles and therapeutic value of these cognitive domains in adolescents with SAD.

## Conclusions

This study examined the role of ToM, metacognitive abilities, and executive functions in adolescents diagnosed with SAD, offering a multidimensional perspective on the cognitive factors that may contribute to the onset or persistence of the disorder. These findings suggest that impairments in working memory and metacognitive beliefs may be particularly useful in differentiating adolescents with SAD from their typically developing peers. When considered together, these cognitive domains represent promising targets for early identification and intervention.

The results underscore the importance of incorporating cognitive assessments particularly those evaluating metacognition and executive functions into both diagnostic and treatment processes for adolescents with SAD. Interventions aimed at improving working memory and modifying dysfunctional metacognitive beliefs may enhance emotional regulation and reduce avoidance behavior, thereby supporting psychological adjustment and improving treatment outcomes. Future research should evaluate the efficacy of such cognitive-based interventions through longitudinal and experimental designs to clarify causal relationships and inform the development of individualized treatment approaches. These findings highlight the relevance of integrating higher-order cognitive functions into clinical models of SAD and may guide the design of adolescent-specific interventions that address the neurocognitive mechanisms contributing to symptom maintenance.

## Data Availability

Data are available from the corresponding author upon reasonable request and subject to approval by the ethics committee or institutional policies.

## Abbreviations

SAD: Social anxiety disorder
TOM: Theory of mind
CAS: Cognitive attention syndrome
K-SADS-PL-DSM-5-T: The Schedule for Affective Disorders and Schizophrenia for School-Age Children Present and Lifetime Version, DSM-5 November 2016 -Turkish Adaptation
WISC-IV: Wechsler intelligence scale for children-fourth edition
Eyes Test: The reading the mind in the eyes test
FPRT: Faux pas recognition test-child version
CNSVS: Cns vital signs
MCQ-C: The metacognition questionnaire for children and adolescents
